# Escalating costs of innovative medicines: perspective and proposals

**DOI:** 10.3389/fpubh.2024.1449707

**Published:** 2024-09-24

**Authors:** Antonio Vallano, Caridad Pontes

**Affiliations:** ^1^Drug Harmonization Program, Medicines Area, Catalan Health Service, Barcelona, Spain; ^2^Corporate Services, Hospital Area, Catalan Institute of Health, Barcelona, Spain; ^3^Department of Pharmacology, Therapeutics and Toxicology, Universitat Autònoma de Barcelona, Barcelona, Spain; ^4^Servei de Farmacologia Clínica, Hospital de la Santa Creu i Sant Pau, Barcelona, Spain

**Keywords:** pharmaceutical costs, national healthcare systems, pricing strategies, regulatory reforms, health-driven payment model

## Abstract

Public healthcare systems are challenged by the soaring costs of medications that require increasing resources, often at the expense of other investments. The increasing pharmaceutical budget poses a threat to the allocation of funds for essential preventive and primary healthcare services while also raising concerns about equitable access, particularly in models where patients bear part of the costs out of their own pockets. Proposals on how to ensure ongoing and long-term accessibility, efficiency, and financial stability are required. The escalating costs of medicines may be explained in part by the mismatch between the traditional value-based pricing and reimbursement frameworks and the type of clinical development of targeted therapies and precision medicine in clinical practice. New appraisal methods and managed access strategies should be adapted to therapies targeting small populations and addressing increased uncertainty. Fair pricing strategies, transparent healthcare investments based on problems and outcomes, regulatory reforms, international cooperation, and critically examining the drug acquisition model are potential solutions. Transitioning from an industry-driven pricing approach to a health-driven payment model can help align the cost of treatments with actual health outcomes, establishing a foundation for a healthcare system that addresses immediate challenges and fosters long-term well-being. Acknowledging the lack of a universally applicable solution, the practical implementation of interventions requires a reframing of the pricing and access system and adaption to the targeted therapeutic approaches. Balancing innovation with financial sustainability necessitates a collaborative, adaptive, and transparent approach, as well as transitioning toward health-driven payment models, moving the focus from the cost of medications to the well-being of populations worldwide.

## 1 Introduction

National health systems face challenges in a complex landscape shaped by ongoing social, environmental, health, scientific, and technological changes (1). The foundation of accessible and affordable healthcare is exhibiting signs of strain, exacerbated by structural and financial challenges (2, 3). The Organization for Economic Co-operation and Development (OECD) data revealed a steady increase in health expenditure from 4.6% of gross domestic product (GDP) in 1970 to 8.8% in 2018, with expectations of continued growth (4). At the core of this concern is the escalating demand for funding, particularly driven by the soaring costs of innovative medicines (5).

Medical science has made remarkable strides, and the regulatory process has made huge advances in accelerating drug access through adaptive regulation (6–12). The unintended consequence is the rising cost of innovative medications, not always commensurate with improvements in health outcomes (13–15). The shift toward specialized drugs for rare diseases or specific indications has further fuelled this cost escalation (16, 17). The increasing investment in medicines challenges the financial robustness of national healthcare systems and compromises the budget allocation for other interventions, questioning their capacity to implement the principles of accessibility, affordability, and sustainability in care (18).

By 2019, pharmaceutical spending became the third-largest component of global healthcare expenditure (19). Although the proportion allocated to medicines in healthcare budgets has remained relatively stable, global medicine spending is projected to reach $1.9 trillion by 2027, growing at a rate of 3–6% per year (20). Notably, in the USA, the costs of the most frequently prescribed brand-name medications for seniors have increased nearly tenfold compared to the annual inflation rate, according to an official report (21). In Spain, pharmacy expenses have grown by 50% in the last 9 years. As the global landscape of pharmaceutical spending continues its upward trajectory, the repercussions are acutely felt within national healthcare systems, posing a significant challenge to healthcare budgets worldwide. This is exemplified by the strain on the United Kingdom’s National Health Service (22).

The pricing of new medicines involves intertwined contributions of both private and public sectors to research and development (R&D) (5, 23). The public sector primarily focuses on foundational research, acting as a catalyst for private sector investments in the discovery and development of medicines. The current model revolves around healthcare systems acquiring goods, where they pay a price that accounts for manufacturing, marketing, and a fair share of the R&D costs of the goods (5, 23).

**Figure 1:**
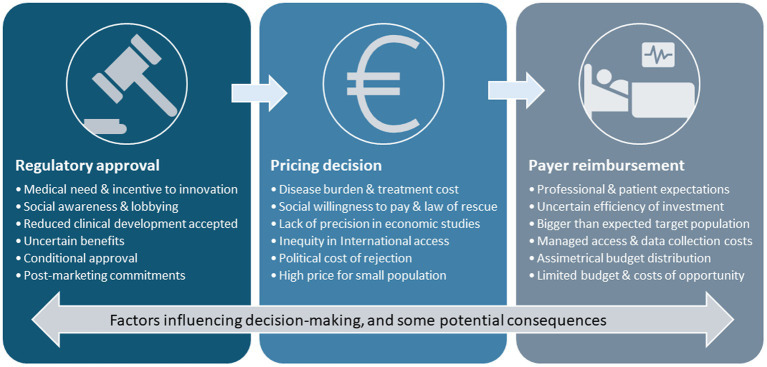
Challenges and complexities in the pharmaceutical landscape. A low prevalence and high-severity medical need are often prioritized as the first indication to apply for commercialization of innovative drugs. Innovation incentives and medical and social awareness of the clinical need and the disease burden emphasize the urge for expediting an early conditional approval that may be based on limited evidence. Once approved, pricing decisions are influenced by uncertainties that prevent building precise economic models, while social need, the rule of rescue, and social willingness to pay tend to accelerate access decisions and determine high prices. Once the product is commercially available, subsequent negotiations for additional indications or competitor products are influenced by the first price, which fails to compensate for the growth of the target population and regulate prices. Professional and patient expectations push payers to prioritize clinical access despite uncertainties and high prices; the relative overpricing leads to asymmetrical budgeting and may represent a high proportion of the healthcare budget at the expense of other investments, thus generating opportunity costs.

The introduction of precision medicine and adaptive marketing authorization plays a transformative role in the ecosystem. The conventional pricing and reimbursement model is designed mostly for the chronic use of drugs by wide populations, based on market dynamics and a predictable return on investment through a stable regulated price of goods paid by healthcare systems. In that framework, highly effective drugs that are used to treat few patients get high prices. The pharmaceutical industry has quickly adapted to the opportunities offered by the new scenario, and the strategic planning of access to products aimed at multiple indications prioritizes the smallest-sized indications with the highest added value to obtain initial exorbitant prices for niche drugs (5). These have paved the way for the negotiations of the subsequent (wider) indications. As the product is already priced and available, its use in new indications may occur before or during the negotiations so that the process may be skewed by increasing impact and frequently results in only marginal price reductions that fail to compensate for the growth of the target population. Furthermore, follow-up drugs intending the same (additional) indications start negotiations at precedent (distorted) prices, progressively departing from the intended value-based pricing approach, thus producing price inflation (Figure 1).

Despite the challenges, the legal framework for pricing and acquisition has remained essentially similar, limiting the room for maneuver of the respective administrations. A fundamental revaluation of the traditional pharmaceutical funding model, acknowledging the rapid growth of medical innovations, clinical uncertainties, and financial challenges, cannot be further delayed. Sellers’ and buyers’ perspectives require realignment to achieve fair pricing, which will preserve sustainability and innovation without compromising access (5, 22, 24, 25). An adaption to an evolving therapeutic paradigm of pricing models, their links to incentives of R&D, and the model of healthcare delivery is required to recover a fair balance between resilient national healthcare systems and the thriving pharmaceutical industry (5, 24, 25). In the following sections, the multifaceted nature of the crisis of escalating pharmaceutical costs, its impact on the sustainability of national healthcare systems, and some comprehensive solutions to specific challenges are summarized.

## 2 Current challenges in global pharmaceutical expenditure: the surge in medication costs and the burden on public healthcare systems

Factors that currently influence innovative drug pricing include those related to return on investment (such as high development costs, amortization of previous failures, dividends and investor’s return, and manufacturing costs), those related to value (such as clinical benefits and health and social impact and perspective), and affordability (such as economic and financial impact, sustainability, and the impact of increasingly higher resources dedicated to acquiring medicines at the expense of other healthcare investments, i.e., opportunity costs). In addition, the consistency with previous decisions (and traceability of the criteria applied) and the influence of the prices of similar products have a substantial impact on the prices. The political and social context determines healthcare policies and priorities, and the prices are influenced by increasing professional and social awareness and expectations regarding innovative drugs, pharmaceutical lobbying power, and the political cost of decisions. The law of supply and demand, the need for innovations, and the availability of supplies to satisfy the demand are relevant determinants of pricing (26–28). Monopoly has emerged as a primary contributor to high drug prices, withholding competition and alternatives (26–28). The high willingness to pay for treatments targeting severe illnesses sustains elevated drug prices (26, 29, 30). It is worth noting that pricing and reimbursement are national competences, which mismatch the global business model of pharmaceutical companies and pose additional difficulties to price setting in each country. Currently, major markets that sustain high-priced innovative medicines create income disparities across diverse markets, and access in low-income countries may not be granted (24). The biopharmaceutical industry justifies that high drug prices are essential for sustaining manufacturing, research, and development (31).

The estimates of drug development costs vary significantly, highlighting methodological differences and the need for transparency in their assessment (5, 32, 33). As companies often rely on public investment for drug discovery, if the public contribution to the discovery is not considered at the time of pricing, society could end up paying twice for the innovation and development of new drugs (34). Furthermore, even if public R&D investment is acknowledged, pricing negotiations lack transparency, with an absence of clarity on how the public investment factors revert to lower overall medication costs (5, 35). In addition, the top 15 biopharmaceutical companies prioritize selling activities over research and development (34).

Regulatory decisions for product authorization and pricing vary, posing challenges in fostering innovation, particularly in less affluent economies (35). Some actors claim that unopposed lobbying, particularly with the European Commission, hinders reforms to reduce drug prices (36, 37). In addition, the lobbying extends to physicians and the public, which obscures visibility, awareness, and advocacy for policies addressing drug costs (26, 38, 39). The challenges posed by orphan drugs and advanced therapies further complicate the landscape, with the limited market for orphan drugs and soaring costs of advanced therapies contributing to a looming crisis for healthcare systems (40–44). Therefore, the current pharmaceutical expenditure model exhibits inherent flaws, with outdated regulations failing to align with evolving therapeutic approaches, leading to negotiations with exorbitant prices for medications that address limited populations (5, 35). The unsustainability of the system is underscored by its failure to consider holistic health outcomes, necessitating a paradigm shift toward proactive healthcare approaches (45).

## 3 Potential solutions: towards a health-driven payment

The current system’s long-term unsustainability requires shifting from a reactive to a proactive approach, which can address root causes rather than merely alleviating symptoms. Revisiting the fundamental concepts of research and development business models, zooming out to the global context, may allow to reconsider the current escalation of prices for achieving fair pricing of medicines (25).

### 3.1 Ensure fair competition

In the context of the upsurge of molecular medicine, the time from discovery to the market has shortened (46), and progressive fractioning of indications has resulted in multiple patents for the same drug and prolonged monopolies (47). The speed of innovation overcomes the time of market protection so that the price regulation role of generics and biosimilars is minimal. In addition, evergreening strategies limit the ability to regulate the market through competition. Thus, reconsideration of the patent system for preventing over-patenting and abuse is one recurrently proposed global strategy (48). Furthermore, imposing stricter penalties would be necessary to discourage “pay-for-delay” schemes, ensuring fair competition (49).

To foster competition, expedited approval processes for generics and biosimilars are essential. Streamlining regulatory approval globally can reduce redundancies but requires trust and cooperation among countries. Non-profit generic manufacturing and compulsory licensing are other avenues for consideration globally, particularly when negotiations for reasonable pricing face obstacles or delays (50, 51). Finally, educating healthcare professionals on biosimilar safety and implementing a comprehensive strategy can facilitate the timely entry of cost-effective biosimilars (52).

### 3.2 Focus on health outcomes

The healthcare value paradigm quantifies enhancements in individual health outcomes relative to the cost incurred. There are already established strategies for managing access based on varying levels of clinical and economic uncertainties. While economic uncertainty tries to contain budgetary impact (e.g., through discounts, tiering, and capping), outcome-based pricing proposes linking drug costs directly to their actual effectiveness in treating specific conditions (53, 54). While outcome-based pricing has been progressively regarded as a potential solution to increasing regulatory uncertainty, its implementation demands a robust framework for measuring health outcomes. Establishing standardized metrics that accurately reflect the effectiveness of treatments has become paramount. Challenges arise in determining value units and fair costs, as well as universally accepted outcome measures. Current standards, such as quality-adjusted life years (QALYs), fall short in considering various pricing determinants. A standardized framework and traceable methodologies for measuring the multiple determinants of health outcomes are necessary, requiring collaboration between healthcare providers, researchers, and pharmaceutical companies (55).

There are also challenges to the implementation of outcome-based models of access, which include resistance from pharmaceutical companies, difficulty in obtaining reliable data on the outcome metrics, and costs of transaction for healthcare providers. Pilot programs may help test and refine outcome metrics and logistics (56, 57). Transfer payments from pharmaceutical firms to payers, when combined with outcome-based pricing, have the potential to enhance financial outcomes for both entities, particularly for drugs with high uncertainties on effectiveness or anticipated low success probabilities (58), but pay-per-performance agreements with products whose health results can be expected in the mid to long term (e.g., gene therapies) require pluriannual implementation periods, which often do not fit into the procurement procedures and mismatch fiscal year-based accounts. In addition, dedicated data collection and periodic assessment of outcomes require huge efforts that are often not foreseen at the inception of agreements and represent the use of public resources to complement missing information on a commercialized product that is paid upfront. We propose that new pricing and reimbursement models are needed to handle conditional authorizations so that the reduced level of evidence at the time of market entry and the public contribution for the clearance of uncertainty are genuinely shared between the industry and healthcare systems (what could be called “pay-per-evidence” models).

### 3.3 Focus on health problems

A paradigm shift from the conventional pricing model (product-based) toward models better suited for new therapeutic approaches (problem-based) should occur to align regulation and pricing frameworks. In such a movement, national healthcare systems should depart from the acquisition of goods to find funding models that may go beyond pharmaceutical product costs, toward a wide view of public health. Payment for health problems involves reimbursing healthcare providers not just for services rendered but for effectively addressing the health problems of the population, thus aligning financial incentives with the resolution of health problems (59–61). However, a standardized classification system, which demands collaborative efforts, for health problems is required, and a profound transformation of the management of health is needed to implement new models that pay per health. Reaching a consensus on categories and standards for pricing may be challenging, and uncertainty regarding incomes may refrain healthcare providers from moving from conventional billing systems based on activity toward outcome-based models. Thorough assessments and pilot programs in especially suited areas may pave the way for effective implementation (62, 63).

### 3.4 Fair negotiation and collaboration

Transparent price and reimbursement negotiations between the ministries of health and pharmaceutical companies have been claimed for long and deemed as an indispensable accountability element. Traceable methods are needed to structure how considerations such as R&D costs, manufacturing costs, clinical value, medical need, and social impact should take precedence when determining a fair price so that pricing and decision-making methodologies become more predictable to all stakeholders (64–67). Multiple criteria decision analysis (MCDA) has been proposed as a systematic approach to collect and organize different elements of pricing and reimbursement decisions (68). Collaborative efforts can produce pricing structures that balance R&D incentives and the financial sustainability of health systems, but this necessarily requires transparency and international coordination. Establishing negotiation platforms and regulatory oversight avoiding information asymmetry may enhance fair and transparent negotiations that are aligned with broader health goals and a fair balance between innovation incentives and financial sustainability (64, 68–71).

### 3.5 Shift to preventive health

Preventive health measures are substantially more effective in improving health and increasing efficiency, and they may alleviate a growing dependence on the use of expensive drugs. Investing in robust preventive and vaccination programs, health education, and lifestyle interventions empowers national health systems to proactively mitigate the financial burden associated with rising pharmaceutical costs but demands an initial investment in a comprehensive strategy and resources; thus, the investment is often delayed due to the lack of prioritization of the required investment for implementing innovation caused by short-term objectives and budget constraints (71). Therefore, determined political support and collaborative efforts between healthcare providers, public health agencies, and educational institutions are required, and a transfer of budgetary resources away from curative measures to preventive interventions is also required (71). Enhanced public awareness of the long-term cost-effectiveness of preventive measures through dedicated campaigns can raise the social demand and political support for preventive healthcare.

### 3.6 Time for regulatory reforms

Governments must consider implementing bold regulatory reforms to encourage fair pricing and increase competition within the pharmaceutical industry. There are repeated demands for tighter and more transparent regulations on pricing practices, patent protection, and market exclusivity to respond and adapt to the changing therapeutic and regulatory background; a new framework is needed to mitigate monopolies contributing to the inflation of drug prices and to create a more competitive landscape (24, 66). Implementing regulatory reforms involves navigating complex legal landscapes and industry and healthcare system dynamics that operate with a long-term view. Governments must strike a delicate balance between ensuring fair pricing and fostering competitive industrial development and the pharmaceutical market through collaboration between legislative bodies, regulatory agencies, and legal experts (35, 64, 68–70). It is key to actively involve the public in discussions to gain support for essential regulatory reforms and to execute a determined implementation plan.

### 3.7 International cooperation

In our globalized pharmaceutical landscape, where borders blur and the interplay between pharmaceutical markets flows, the lack of international cooperation between governments, regulatory bodies, and pharmaceutical companies exacerbates the regulatory challenges on drug pricing and access. Individual national policies add layers of complexity, hampering consistency and cohesion (35). Addressing the rising medication costs demands collaboration globally, the exploration of innovative solutions aligned with a common global framework, and the avoidance of threats that may derive from conceptually heterogeneous criteria across markets.

Recognizing the global nature of pharmaceutical markets, international cooperation has emerged as a linchpin for any transformative change. International agreements and regulatory standards for fair pricing demand collaboration between governments, international organizations, and the pharmaceutical industry. There are many barriers to the cooperation, such as different healthcare models and priorities, economic wealth differences among nations, national industrial interests, and ensuring compliance with international agreements and standards (72, 73). Diplomatic efforts for aligning global healthcare priorities and establishing an international body to oversee and enforce fair pricing practices may help overcome these barriers, and a global consensus on ethical pricing practices is paramount.

### 3.8 Effective implementation

The long cycles of the current models of incentives, research, pricing and return on investment, and thus the time for implementation cannot be short-term, especially considering that the very essence of paying per goods may need to be revisited. Such a movement requires a collaborative and progressive plan navigating complex regulatory environments that allow to overcome the inertia of decades while addressing barriers and resistances from different stakeholders. All the processes should ensure that the changes benefit healthcare, incentives industry, and thus require careful consideration and strategic planning. Piloting of specific solutions to the newly appeared issues may allow a progressive and practical implementation, paralleling regulatory adaptions that recognize that a one-size-fits-all solution does not exist.

The Pharmaceutical Strategy for Europe and associated proposals for reforms contain proposals for the adaption of a regulatory setting that opens a valuable window of opportunity for advancement (35, 74). Many potential solutions may be incorporated into the regulations, such as those summarized in Table 1, where various specific problems and possible solutions for addressing different challenges in drug pricing and reimbursement are proposed. Some are already in practice, while some are still theoretical and may be helpful for advancing in the transformation, but require development. These could be piloted to learn about their performance and implementation barriers and facilitators. Tailoring these strategies to the unique context of each healthcare system may allow a balance between the imperative for innovation and the need for financial sustainability.

**Table 1:** Some challenges in the drug pricing and reimbursement of medicines, and potential solutions.

Challenge	Problem	Potential solution
Conditional approvals	Social claim for early access to innovation for unmet needs and uncertainty in clinical and economic models make it difficult to set the value and price. Once approved, it is difficult to withdraw or revisit the price even if evidence shows lower effectiveness than expected. Setting of disproportionate prices per value as the reference. No compensation for opportunity costs of regulatory failures if new drugs do not confirm risk–benefit.	“Pay-per-evidence” models: Based on a theoretical final price set according to the best clinical expectations, apply N conditional discounts accounting for uncertainties (missing information) at the time of market entry, and proportional to the missing information. Conditional discounts can be linked to regulatory conditions and/or additional conditions. As conditions are met, conditional discounts can be progressively removed.
Authorization under exceptional circumstances. Treatments with single administration and expected long-term effect	The development of ATMP for rare conditions is limited due to a reduced target population, and the disappearance of the potential market if large trials are conducted. The product price is not only extremely high due to reduced target and potentially high value but is also highly uncertain. Single down payment for a treatment is a high cash burden and can become a barrier to access. “Mortgage-like” agreements based on outcomes pose transaction costs and difficult cash-flow and taxes management. Access can be managed through pay-per-performance agreements but can be difficult to budget and purchase.	Delink incentives to R&D from healthcare costs, and set prices related to manufacturing and delivering costs. Explore public–private partnerships to complete evidence generation for late clinical development. Shared development can include milestone payments at proof of concept and fairly shared costs for confirmatory development, including costs of goods and costs of data collection.
Orphan drugs and very small target populations	There is a European mandate on equitable drug access, but high prices may not be affordable across different healthcare systems and economies. Social willingness to pay for severe diseases and the rule of rescue increase the pressure and political costs of negotiation. Companies avoid big differences in price across countries and withdraw P&R applications to avoid price erosion.	Delink incentives to R&D from sales. Manage equity by setting the prices using GNP-weighed cost-effectiveness thresholds. Regulate early access before P&R to incentivize realistic negotiation. Avoid dedicated funds that may encourage funding of low-value products.
Early individual access and compassionate use	Passing mechanisms for P&R control may lead to systematic access in rare unmet needs. Early access at foreign prices higher than those traditionally given may hamper negotiation or even discourage commercialization. Healthcare professionals, scientific societies, patients, and media do claim early access, which increases the pressure and political costs of negotiation.	Regulate conditions of early access before P&R decision (product at no cost or low prices). Incentivize negotiation through the regularization of early-access costs, i.e., if the product is commercialized, the agreed prices and reimbursement conditions are to be applied retrospectively once the P&R process is completed. If an agreement is not reached and the product is rejected, the product costs of the started treatments should be covered by the marketing authorization holder.
Additional indications for a commercialized product	Too-high initial prices, the weakness of negotiation with the product in the market, and value-based prices as mixed cases.	Review regulation to include automated pricing revision based on sales. Improve the methodology for budget impact estimation and the use of maximum-level models of sales-tiered prices.
Add-on treatments and drug combinations	Initial approval of a drug as monotherapy in the late lines of therapy with prices as in orphan setting, followed by earlier stage indication as an add-on to the standard of care or drug combination. Often N medicines from different manufacturers involved, making negotiation difficult. Initial prices set at a high cost add to the standard of care, increasing the costs of treating the disease.	Develop methods to set a maximum cost per patient per disease. Set thresholds for price based on disease costs. Review regulation to force pricing review of already commercialized drugs involved in combined schedules based on disease costs.
Patent evergreening	Chained strategies for life-cycle management take maximum profit of exclusivity rights, delaying competition and self-regulation. Small target populations may be captured, e.g., through trials or open-access programs at no cost for new formulations or posology, blocking competition. Voluntary lowering of prices by an originator that can afford temporary economic loss due to previous benefits, acting as barriers to biosimilar products entering the market.	Revisit exclusivity rights, and set limits to monopolies according to the degree of innovation. Revisit laws of competition, and set obligations to licensing. Implement specific P&R strategies and policies that encourage competition as original medicines approach the end of the exclusivity period. Incentives for companies offering affordable and effective alternatives.
Availability of antimicrobial medicines and essential medicines	Medical need requires new antibiotics to be available but are seldom used. Pricing and return on investment based on the volume of sales discourage investment.	Delink incentives to R&D from sales, and explore public–private R&D models. Payment models may include down payment per milestones, flat fees per availability, or tiered prices based on sales.

P&R, Pricing and reimbursement; R&D, Research and development; ATMP, Advanced therapy medicinal product; GNP, Gross national product.

## 4 Conclusion

From the inherent flaws in the current pharmaceutical expenditure model to the global complexities of regulatory frameworks, a comprehensive understanding of the challenges of escalating medication costs is essential. Some challenges and strains of the current models are highlighted by the cases of orphan drugs and advanced therapies, and potential solutions to address them include a global compromise for fair pricing, fostering innovation, and ensuring the financial sustainability of national healthcare systems.

Acknowledging the absence of a universally applicable solution, the practical implementation of targeted interventions necessitates a tailored approach for each healthcare system, which may be guided by the progressive piloting of new models. It may require a fundamental reform of the current pharmaceutical model, ensuring not only R&D incentives but also that healthcare remains accessible, efficient, and financially sustainable in the long-term. Immediate challenges to healthcare systems can be addressed by exploring alternative pricing strategies based on health problems and outcomes, freeing resources for preventive measures aimed at improving the long-term well-being of the global population. Fostering negotiation and international cooperation is needed to align international strategies with national competences.

Prospective planning and a collaborative, adaptive approach may allow to progress toward a healthcare future where costs are fairly proportional to outcomes, focusing on the well-being of the population and maintaining incentives for a vibrant and flourishing innovation.

## Data Availability

The original contributions presented in the study are included in the article/supplementary material, further inquiries can be directed to the corresponding author.

## References

[1] Khan Z. The emerging challenges and strengths of the national health services: a physician perspective. Cureus. (2023) 15:e38617. doi: 10.7759/cureus.38617, PMID: 37284412 PMC10240167

[2] Giovanella L Stegmüller K. The financial crisis and health care systems in Europe: universal care under threat? Trends in health sector reforms in Germany, the United Kingdom, and Spain. Cad Saude Publica. (2014) 30:2263–81. doi: 10.1590/0102-311x00021314 25493982

[3] Palasca S Jaba E. Economic crisis' repercussions on European healthcare systems. Proc Econ Finance. (2015) 23:525–33. doi: 10.1016/S2212-5671(15)00568-7

[4] OECD. Health at a glance 2019: OECD indicators. Paris: OECD Publishing (2019).

[5] Morgan SG Bathula HS Moon S. Pricing of pharmaceuticals is becoming a major challenge for health systems. BMJ. (2020) 368:l4627. doi: 10.1136/bmj.l4627, PMID: 31932289

[6] Mukherjee S. The emperor of all maladies: a biography of cancer. New York: Scribner (2010).

[7] Dunbar CE High KA Joung JK Kohn DB Ozawa SM Sadelain M. Gene therapy comes of age. Science. (2018) 359:eaan4672. doi: 10.1126/science.aan4672 29326244

[8] Esfahani K Roudaia L Buhlaiga N Del Rincon SV Papneja N Miller WH. A review of cancer immunotherapy: from the past, to the present, to the future. Curr Oncol. (2020) 27:87–97. doi: 10.3747/co.27.5223, PMID: 32368178 PMC7194005

[9] Ginsburg GS Phillips KA. Precision medicine: from science to value. Health Aff (Millwood). (2018) 37:694–701. doi: 10.1377/hlthaff.2017.1624, PMID: 29733705 PMC5989714

[10] Martínez-Espinosa R Ramírez-Vélez G. mRNA-based COVID-19 vaccines: a new age. Multidis J Healthc. (2021) 1:18–30. doi: 10.36105/psrua.2021v1n2.03

[11] Zheng R Zhang L Parvin R Su L Chi J Shi K. Progress and perspective of CRISPR-Cas9 technology in translational medicine. Adv Sci (Weinh). (2023) 10:e2300195. doi: 10.1002/advs.202300195, PMID: 37356052 PMC10477906

[12] Eichler HG Baird LG Barker R Bloechl-Daum B Børlum-Kristensen F Brown J. From adaptive licensing to adaptive pathways: delivering a flexible life-span approach to bring new drugs to patients. Clin Pharmacol Ther. (2015) 97:234–46. doi: 10.1002/cpt.59, PMID: 25669457 PMC6706805

[13] Xu S Kesselheim AS. Medical innovation then and now: perspectives of innovators responsible for transformative drugs. J Law Med Ethics. (2014) 42:564–75. doi: 10.1111/jlme.12176, PMID: 25565621

[14] Shin G Kwon HY Bae S. For whom the price escalates: high price and uncertain value of cancer drugs. Int J Environ Res Public Health. (2022) 19:4204. doi: 10.3390/ijerph19074204, PMID: 35409887 PMC8998346

[15] Nieto-Gómez P Castaño-Amores C Rodríguez-Delgado A Álvarez-Sánchez R. Analysis of oncological drugs authorised in Spain in the last decade: association between clinical benefit and reimbursement. Eur J Health Econ. (2023) 25:257–67. doi: 10.1007/s10198-023-01584-9, PMID: 36995531

[16] Sharma A Jacob A Tandon M Kumar D. Orphan drug: development trends and strategies. J Pharm Bioallied Sci. (2010) 2:290–9. doi: 10.4103/0975-7406.72128, PMID: 21180460 PMC2996062

[17] Gibson SG Lemmens T. Niche markets and evidence assessment in transition: a critical review of proposed drug reforms. Med Law Rev. (2014) 22:200–20. doi: 10.1093/medlaw/fwu005, PMID: 24841527

[18] Tichy EM Hoffman JM Suda KJ Rim MH Tadrous M Cuellar S. National trends in prescription drug expenditures and projections for 2022. Am J Health Syst Pharm. (2022) 79:1158–72. doi: 10.1093/ajhp/zxac102, PMID: 35385103 PMC9383648

[19] OECD. OECD Health at a glance 2021: OECD indicators. Paris: OECD Publishing (2021).

[20] IQVIA. The global use of medicine in 2019 and outlook to 2023. (2022). Available at: https://www.iqvia.com/insights/the-iqvia-institute/reports-and-publications/reports/the-global-use-of-medicines-2023 (Accessed December 6, 2023).

[21] Homeland Security & Governmental Affairs. Breaking: brand-name drugs increasing at 10x cost of inflation, Mccaskill report finds (2018). Available at: https://www.hsgac.senate.gov/media/dems/breaking-brand-name-drugs-increasing-at-10x-cost-of-inflation-mccaskill-report-finds/ (Accessed December 6, 2023).

[22] Simoens S Huys I. How much do the public sector and the private sector contribute to biopharmaceutical R&D? Drug Discov Today. (2022) 27:939–45. doi: 10.1016/j.drudis.2021.11.027, PMID: 34863932

[23] Bennett SR. Changes in healthcare during the past 30 years: can the national health service in the United Kingdom survive? Cureus. (2023) 15:e38120. doi: 10.7759/cureus.38120, PMID: 37252534 PMC10212742

[24] Moon S Mariat S Kamae I Pedersen HB. Defining the concept of fair pricing for medicines. BMJ. (2020) 368:l4726. doi: 10.1136/bmj.l4726, PMID: 31932334

[25] Suleman F Low M Moon S Morgan SG. New business models for research and development with affordability requirements are needed to achieve fair pricing of medicines. BMJ. (2020) 368:l4408. doi: 10.1136/bmj.l4408, PMID: 31932324

[26] Rajkumar SV. The high cost of prescription drugs: causes and solutions. Blood Cancer J. (2020) 10:71. doi: 10.1038/s41408-020-0338-x, PMID: 32576816 PMC7311400

[27] Siddiqui M Rajkumar SV. The high cost of cancer drugs and what we can do about it. Mayo Clinic Proc. (2012) 87:935–43. doi: 10.1016/j.mayocp.2012.07.007, PMID: 23036669 PMC3538397

[28] Kantarjian H Rajkumar SV. Why are cancer drugs so expensive in the United States, and what are the solutions? Mayo Clinic Proc. (2015) 90:500–4. doi: 10.1016/j.mayocp.2015.01.014, PMID: 25792242

[29] Cookson R McCabe C Tsuchiya A. Public healthcare resource allocation and the rule of rescue. J Med Ethics. (2008) 34:540–4. doi: 10.1136/jme.2007.021790, PMID: 18591290

[30] Hlatky MA. Willingness to pay for high-cost medications. Circulation. (2020) 141:1225–6. doi: 10.1161/CIRCULATIONAHA.120.045966 32282251

[31] DiMasi JA Grabowski HG Hansen RW. Innovation in the pharmaceutical industry: new estimates of R&D costs. J Health Econ. (2016) 47:20–33. doi: 10.1016/j.jhealeco.2016.01.012 26928437

[32] Schalander M Hernadez-Villafuerte K Cheng CY Mestre-Ferrandiz J Bauman M. How much does it cost to research and develop a new drug? A systematic review and assessment. Pharmacoeconomics. (2021) 39:1243–69. doi: 10.1007/s40273-021-01065-y, PMID: 34368939 PMC8516790

[33] Wouters OJ McKee M Luyten J. Estimated research and development investment needed to bring a new medicine to market 2009-2018. JAMA. (2020) 323:844–53. doi: 10.1001/jama.2020.1166, PMID: 32125404 PMC7054832

[34] Angelis A Polyakov R Wouters OJ Torreele E McKee M. High drug prices are not justified by industry's spending on research and development. BMJ. (2023) 380:e071710. doi: 10.1136/bmj-2022-071710 36792119

[35] European Commission. A pharmaceutical strategy for Europe. (2023). Available at: https://health.ec.europa.eu/medicinal-products/pharmaceutical-strategy-europe_en (Accessed April 24, 2024).

[36] Scuti S. Big pharma spends record millions on lobbying amid pressure to lower drug prices. Cable News Network – CNN Health. (2019). Available at: https://edition.cnn.com/2019/01/23/health/phrma-lobbying-costs-bn/index.html (Accessed April 24, 2024).

[37] Corporate Europe Observatory. High prices, poor access: the EU medicines market and big pharma. What is big pharma fighting for in Brussels? Corporate Europe Observatory. (2019). Available at: https://corporateeurope.org/en/2019/05/high-prices-poor-access-eu-medicines-market-and-big-pharma (Accessed April 24, 2024).

[38] Lexchin J. Interactions between physicians and the pharmaceutical industry: what does the literature say? CMAJ. (1993) 149:1401–7. PMID: 8221424 PMC1485922

[39] McGuire C King S Roche-Nagle G Barry MC. Doctors' attitudes about prescribing and knowledge of the costs of common medications. Ir J Med Sci. (2009) 178:277–80. doi: 10.1007/s11845-009-0276-x, PMID: 19221833

[40] Carr DR Bradshaw SE. Gene therapies: the challenge of super-high-cost treatments and how to pay for them. Regen Med. (2016) 11:381–93. doi: 10.2217/rme-2016-0010, PMID: 27185544

[41] Simoens S De Groote K Boersma C. Critical reflections on reimbursement and access of advanced therapies. Front Pharmacol. (2022) 13:771966. doi: 10.3389/fphar.2022.771966, PMID: 35662719 PMC9157586

[42] Connock M Andronis L Auguste P Dussart C Armoiry X. Will the US$5 million onasemnogene abeparvosec treatment for spinal muscular atrophy represent ‘value for money’ for the NHS? A rapid inquiry into suggestions that it may be cost-effective. Expert Opin Biol Ther. (2020) 20:823–7. doi: 10.1080/14712598.2020.1772747, PMID: 32434404

[43] Cook K Forbes SP Adamski K Ma JJ Chawla A Garrison LP. Assessing the potential cost-effectiveness of a gene therapy for the treatment of hemophilia A. J Med Econ. (2020) 23:501–12. doi: 10.1080/13696998.2020.1721508, PMID: 31971453

[44] Iglesias-López C Agustí A Vallano A Obach M. Financing and reimbursement of approved advanced therapies in several European countries. Value Health. (2023) 26:841–53. doi: 10.1016/j.jval.2022.12.014 36646280

[45] Gonçalves E. Value-based pricing for advanced therapy medicinal products: emerging affordability solutions. Eur J Health Econ. (2022) 23:155–63. doi: 10.1007/s10198-021-01276-2, PMID: 34106364 PMC8882079

[46] Beall RF Hwang TJ Kesselheim AS. Pre-market development times for biologic versus small-molecule drugs. Nat Biotechnol. (2019) 37:708–11. doi: 10.1038/s41587-019-0175-2, PMID: 31213674

[47] Feldman R. May your drug price be evergreen. J Law Biosci. (2018) 5:590–647. doi: 10.1093/jlb/lsy022, PMID: 31143456 PMC6534750

[48] Siddalingaiah S Fugh-Berman A. Evergreened drugs or evergreened profits? J Eval Clin Pract. (2022) 28:1119–26. doi: 10.1111/jep.13695, PMID: 35543377

[49] Hancock J Lupkin S. Secretive ‘rebate trap’ keeps generic drugs for diabetes and other ills out of reach. Kaiser Health News. (2019). Available at: https://abcnews.go.com/Health/secretive-rebate-trap-generic-drugs-diabetes-ills-reach/story?id=60490771 (Accessed April 24, 2024).

[50] Betz M. The new nonprofit pharmaceutical world: what’s up with that? (2018). Available at: https://nonprofitquarterly.org/the-new-nonprofit-pharmaceutical-world-whats-up-with-that/ (Accessed December 7, 2023).

[51] Kerry VB Lee K. TRIPS, the Doha declaration and paragraph 6 decision: what are the remaining steps for protecting access to medicines? Glob Health. (2007) 3:3. doi: 10.1186/1744-8603-3-3, PMID: 17524147 PMC1892549

[52] Godman B Tubic B Allocati E Wladysiuk M McTaggart S Kurdi A. Biosimilars are essential for sustainable healthcare systems; however, key challenges remain as seen with long-acting insulin analogues. J Appl Pharm Sci. (2022) 12:55–072. doi: 10.7324/JAPS.2022.120306

[53] Campbell JD Kaló Z. Fair global drug pricing. Expert Rev Pharmacoecon Outcomes Res. (2018) 18:581–3. doi: 10.1080/14737167.2018.1524296 30215535

[54] Vlaanderen FP Tanke MA Bloem BR Faber MJ Eijkenaar F Schut FT. Design and effects of outcome-based payment models in healthcare: a systematic review. Eur J Health Econ. (2019) 20:217–32. doi: 10.1007/s10198-018-0989-8, PMID: 29974285 PMC6438941

[55] Bohm N Bermingham S Grimsey Jones F Gonçalves-Bradley DC Diamantopoulos A Burton JR. The challenges of outcomes-based contract implementation for medicines in Europe. PharmacoEconomics. (2022) 40:13–29. doi: 10.1007/s40273-021-01070-1, PMID: 34480324 PMC8738500

[56] Carlson JJ Sullivan SD Garrison LP Neumann PJ Veenstra DL. Linking payment to health outcomes: a taxonomy and examination of performance-based reimbursement schemes between healthcare payers and manufacturers. Health Policy. (2010) 96:179–90. doi: 10.1016/j.healthpol.2010.02.005, PMID: 20226559

[57] Carlson JJ Gries KS Yeung K Sullivan SD Garrison LP Jr. Current status and trends in performance-based risk-sharing arrangements between healthcare payers and medical product manufacturers. Appl Health Econ Health Policy. (2014) 12:231–8. doi: 10.1007/s40258-014-0093-x, PMID: 24664994

[58] Adida E. Outcome-based pricing for new pharmaceuticals via rebates. Manag Sci. (2020) 67:892–913. doi: 10.1287/mnsc.2019.3574

[59] Teisberg E Wallace S O'Hara S. Defining and implementing value-based health care: a strategic framework. Acad Med. (2020) 95:682–5. doi: 10.1097/ACM.0000000000003122, PMID: 31833857 PMC7185050

[60] Steenhuis S Struijs J Koolman X Ket J Van der Hijden E. Unraveling the complexity in the design and implementation of bundled payments: a scoping review of key elements from a payer's perspective. Milbank Q. (2020) 98:197–222. doi: 10.1111/1468-0009.12438, PMID: 31909852 PMC7077767

[61] Bour SS Raaijmakers LHA Bischoff EWMA Goossens LMA Rutten-van Mölken MPMH. How can a bundled payment model incentivize the transition from single-disease management to person-centred and integrated care for chronic diseases in the Netherlands? Int J Environ Res Public Health. (2023) 20:3857. doi: 10.3390/ijerph20053857, PMID: 36900870 PMC10001506

[62] Guler J Roberts MC Medina-Mora ME Robles R Gureje O Keeley JW. Global collaborative team performance for the revision of the international classification of diseases: a case study of the World Health Organization field studies coordination group. Int J Clin Health Psychol. (2018) 18:189–200. doi: 10.1016/j.ijchp.2018.07.001, PMID: 30487924 PMC6224857

[63] Kruk ME Gage AD Arsenault C Jordan K Leslie HH Roder-DeWan S. High-quality health systems in the sustainable development goals era: time for a revolution. Lancet Glob Health. (2018) 6:e1196–252. doi: 10.1016/S2214-109X(18)30386-3, PMID: 30196093 PMC7734391

[64] Elvira D Torres F Vives R Puig G Obach M Gay D. Reporting reimbursement price decisions for onco-hematology drugs in Spain. Front Public Health. (2023) 11:1265323. doi: 10.3389/fpubh.2023.1265323, PMID: 37942255 PMC10627880

[65] International Association of Mutual Benefit Societies. AIM proposes to establish a European drug pricing model for fair and transparent prices for accessible pharmaceutical innovations. (2019). Available at: https://www.aim-mutual.org/wp-content/uploads/2019/12/AIMfairpricingModel.pdf (Accessed April 24, 2024).

[66] Kaltenboeck A Bach PB. Value-based pricing for drugs: theme and variations. JAMA. (2018) 319:2165–6. doi: 10.1001/jama.2018.4871 29710320

[67] La Rosa F Liberatore G. Biopharmaceutical and chemical firms’ R&D disclosure, and cost of equity: the impact of the regulatory regime. Eur Manag J. (2014) 32:806–20. doi: 10.1016/j.emj.2014.01.003

[68] Elvira D Obach M Pontes C. Description of the use of multicriteria to support pricing and reimbursement decisions by European health technology assessment bodies. BMC Health Serv Res. (2021) 21:814. doi: 10.1186/s12913-021-06784-8, PMID: 34391431 PMC8364048

[69] World Health Organization. Regional Office for Europe. Challenges and opportunities in improving access to medicines through efficient public procurement in the WHO European region. World Health Organization Regional Office for Europe. (2016). Available at: https://iris.who.int/handle/10665/344000 (Accessed April 24, 2024).

[70] Rieg R Vanini U. Value relevance of voluntary intellectual capital disclosure: a meta-analysis. Rev Manag Sci. (2023) 17:2587–631. doi: 10.1007/s11846-023-00630-3

[71] Masters R Anwar E Collins B Cookson R Capewell S. Return on investment of public health interventions: a systematic review. J Epidemiol Community Health. (2017) 71:827–34. doi: 10.1136/jech-2016-208141 28356325 PMC5537512

[72] World Health Organization. WHO guideline on country pharmaceutical pricing policies. 2nd ed. Geneva: World Health Organization (2020). 33950613

[73] Vidal J. Report medicine pricing and access in Europe and beyond. Health Action International. (2021). Available at: https://haiweb.org/wp-content/uploads/2021/11/HAI-Policy-Report-Medicine-Pricing.pdf (Accessed April 24, 2024).

[74] European Commission. Reform of the EU pharmaceutical legislation. Affordable, accessible, and innovative medicines. (2023). Available at: https://commission.europa.eu/strategy-and-policy/priorities-2019-2024/promoting-our-european-way-life/european-health-union/reform-eu-pharmaceutical-legislation_en (Accessed April 24, 2024).

